# Differences in functional connectivity distribution after transcranial direct‐current stimulation: A connectivity density point of view

**DOI:** 10.1002/hbm.26112

**Published:** 2022-11-13

**Authors:** Bohao Tang, Yi Zhao, Archana Venkataraman, Kyrana Tsapkini, Martin A. Lindquist, James Pekar, Brian Caffo

**Affiliations:** ^1^ Department of Biostatistics Johns Hopkins University Baltimore Maryland USA; ^2^ Department of Biostatistics and Health Data Science Indiana University School of Medicine Indianapolis Indiana USA; ^3^ Department of Electrical and Computer Engineering Johns Hopkins University Baltimore Maryland USA; ^4^ Department of Neurology Johns Hopkins Medicine Baltimore Maryland USA; ^5^ Department of Cognitive Science Johns Hopkins Medicine Baltimore Maryland USA; ^6^ F.M. Kirby Research Center for Functional Brain Imaging Kennedy Krieger Institute Baltimore Maryland USA; ^7^ Department of Radiology and Radiological Science Johns Hopkins University Medicine Baltimore Maryland USA

**Keywords:** density regression, functional connectivity, random graph

## Abstract

In this manuscript, we consider the problem of relating functional connectivity measurements viewed as statistical distributions to outcomes. We demonstrate the utility of using the distribution of connectivity on a study of resting‐state functional magnetic resonance imaging association with an intervention. The method uses the estimated density of connectivity between nodes of interest as a functional covariate. Moreover, we demonstrate the utility of the procedure in an instance where connectivity is naturally considered an outcome by reversing the predictor/response relationship using case/control methodology. The method utilizes the density quantile, the density evaluated at empirical quantiles, instead of the empirical density directly. This improved the performance of the method by highlighting tail behavior, though we emphasize that by being flexible and non‐parametric, the technique can detect effects related to the central portion of the density. To demonstrate the method in an application, we consider 47 primary progressive aphasia patients with various levels of language abilities. These patients were randomly assigned to two treatment arms, transcranial direct‐current stimulation and language therapy versus sham (language therapy only), in a clinical trial. We use the method to analyze the effect of direct stimulation on functional connectivity. As such, we estimate the density of correlations among the regions of interest and study the difference in the density post‐intervention between treatment arms. We discover that it is the tail of the density, rather than the mean or lower order moments of the distribution, that demonstrates a significant impact in the classification. The new approach has several benefits. Among them, it drastically reduces the number of multiple comparisons compared with edge‐wise analysis. In addition, it allows for the investigation of the impact of functional connectivity on the outcomes where the connectivity is not geometrically localized.

## Graphical abstract

In this manuscript, we develop a method utilizing the distribution of resting‐state fMRI connectivity measurements to study the effect of a direct stimulation. The method can drastically reduce the number of multiple comparisons compared with edge‐wise analysis. In addition, it allows for the investigation of the impact of functional connectivity on the outcomes where the connectivity is not geometrically localized.

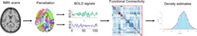

## 1 INTRODUCTION

The study of resting‐state brain connectivity via functional magnetic resonance imaging (fMRI) involves the investigation of correlations between cortical seeds, regions, or voxels (henceforth referred to as foci). Friston, in particular, defined functional connectivity as the correlations, over time, between spatially distinct brain regions (Friston, 2011). Nearly all inter‐subject investigations of connectivity have focused on *localized correlations*. That is, they consider correlations between foci treated consistently across subjects. Mathematically, this can be described as saying that the methods are not invariant to subject‐specific relabeling of the foci. In fact, for most methods, such as pairwise regressions on correlations across subjects or decomposition methods, shuffling foci labels within subjects is a form of null distribution. Furthermore, this lack of invariance applies regardless of the degree of granularity of the analysis, from regions to seeds to voxels (Bastos & Schoffelen, 2016; Damoiseaux & Greicius, 2009; Friston, 2011). The methods and choice of granularity all center the focus on geographic consistency of correlations across groups of similar subjects. Individual topography (Kong et al., 2019) and functional connectivity alignment (Haxby et al., 2020) are another set of methods that allow for spatially inconsistent relationships beyond subject‐specific structure. However, their effort of finding subject‐specific parcellation/transformation is still for the purpose of localization. Other exceptions include many variations of graph theory‐based methods, where graphical features may not be localized across subjects in the sense of summarizing multiple connections (Shen et al., 2017) or being invariant to subject‐specific foci labels (Koutra et al., 2013; Vogelstein et al., 2012).

To illustrate the idea of label invariance, consider a scenario where one reduces the connectivity measures to subject‐specific binary graphs (by thresholding). If the effect of the graphs on the outcomes is invariant to the nodes (foci) corresponding to the edges, then clearly it is sufficient to know the number of edges that are present for each subject's graph, since given that information one can create the set of equivalent graphs under node invariance. This is equivalent to saying the relationship between the outcome and connectivity graph, is solely dependent on the estimated probability distribution for the edges under an assumed independent and identically distributed edge distribution, since that distribution only depends on the total number of edges. (This is the Erdős‐Rényi random graph model.) Our approach formally builds on this idea. But we further consider a random weighted graph model rather than thresholding to obtain binary edges, and propose a specific functional linear model for the relationship between outcomes and the connectivity density.

We demonstrate the benefits of using the distribution of resting state correlations as covariates using functional data analysis tools. We suggest the use of the quantile density, the density of connections evaluated at evenly spaced quantiles of the connections, as this improves performance. Regardless of these choices, utilizing connectivity density regression has several benefits. A primary one is the relaxation of the consistent localization assumption across subjects. In the Appendix A, we demonstrate mathematically how connection densities achieve this invariance. Localization analyses make the, often unchallenged, assumption that pairs of foci represent the same correlated functional specialization across exchangeable subjects. This assumption is grounded in the neurological theory of functional specialization dating back to the foundational works of Broca and Weirnicke (Broca, 1861; Wernicke, 1874). However, it is clear that in specific applications and biological settings, the neural geography of functional specialization can vary. As an extreme example, subjects with brain damage in their youth often have the neuroplasticity that remaps a function to atypical areas (Finger & Almli, 1985).

Hyperalignment (Haxby et al., 2020) also allows for a high degree of subject‐specific functional specialization. However, unlike connectivity density regression, localization remains the goal in hyperalignment, and therefore, a multiparameter alignment transformation must be estimated per subject. Connectivity density analysis can be seen as a complementary, technique that does not require the estimation of subject‐specific alignment. Further, focusing on connectivity densities drastically simplifies the problem and reduces multiplicity concerns. Of course, these benefits come at the cost of not considering potentially relevant localization information, and so the technique cannot be more sensitive to the detection of localized effects with a reduced search space and correct a priori localization hypotheses. It would be accurate to say that focusing on connectivity densities in analysis lies at one end of the spectrum of model localization assumptions, whereas pair at a time models lie at the other extreme and hyperalignment lying somewhere in the middle.

There are existing studies that utilize the distribution of resting state correlations. For example, Petersen and Muüller (2016) consider the distribution of correlations between a seed voxel and all other voxels within regions of interest (ROI), to summarize the ROI state. Also, Scheinost et al. (2012) further considered such distributions across all pairs of voxels. This work derived a degree function from the connection density as a summary of the connectivity of each voxel. As a result, these studies continue to focus on localized effects, where the use of the connectivity density is mainly to achieve a more informative localized summary of brain connectivity.

This study is motivated by a resting‐state fMRI study of primary progressive aphasia (PPA) patients, where it is feasible to want to relax the geometric localization assumption. In the study, the patients were randomly assigned into two treatment groups, (a) transcranial direct‐current stimulation (tDCS; Nitsche et al., 2008) and language therapy versus (b) a sham tDCS and language therapy only. In the tDCS group, the nominal stimulation target was the left inferior frontal gyrus (IFG). Since the actual area of stimulation may vary, even if only slightly, it is relevant to consider models that are less dependent on localization. In addition, the stimulation electrode patches were size of 5 × 5 = 25 cm^2^. Thus, the stimulation areas may have extended beyond the left IFG in a way that may induce additional variation across subjects that would also motivate considering techniques that are robust to violations of localization assumptions. Here, we propose a novel approach to represent the effect of stimulation on functional connectivity. By ignoring spatial heterogeneity, we directly study the change on the distribution of correlation between the ROIs.

The manuscript is organized as follows. In Section 2, the experimental design and approach are introduced. Results both for simulated and real data are shown in Section 3. Section 4 contains a summary and discussion.

## 2 MATERIAL AND METHODS

### 2.1 Experimental design

The data analyzed in this study were part of a larger randomized, double‐blinded, sham‐controlled, crossover study on aphasia treatment using tDCS. All of the analyzed subjects had at least 2 years of progressive language deficit and no history of any other neurological condition that may have affected their language ability. Subjects had atrophy predominantly in the left hemisphere. Subjects were diagnosed via neuropsychological testing, language testing, MRI, and clinical assessment according to consensus criteria (Gorno‐Tempini et al., 2011). The study was approved by the Johns Hopkins Hospital Institutional review board and all subjects provided informed consent to participate in the study.

Each subject was diagnosed with one of the PPA variant types: logopenic, nonfluent, or semantic. Randomization was conducted within each variant type with an equal probability assigned to either the tDCS or sham group. As shown in Table 1, the two groups are balanced in both demographic and clinical characteristics. The language component of severity was evaluated based on the revised fronto‐temporal dementia clinical dementia rating (FTD‐CDR) used to rate severity in PPA (Knopman et al., 2008). To calculate severity, three raters independently scored each item based on the interaction with the participant and family, language, cognitive testing, and questionnaires, followed by a discussion to produce a consensus score. In the tDCS group, the Soterix Transcranial Direct Current Stimulation 1 × 1 Clinical Trials device (Model 1500) was used to deliver tDCS (for tDCS setup details, see Tsapkini et al., 2018). The anode was placed over the left frontal lobe and the cathode was placed over the right cheek. The size of the nonmetallic, conductive rubber electrodes (fitted with saline‐soaked sponges to limit skin‐electrode reactions) is 5 cm × 5 cm, which covers the whole left IFG. In each tDCS session, the density of the delivered current was 2 mA and the delivery lasted for 20 min. Simultaneous with the tDCS delivery, language therapy was initiated and continued for an additional 20–25 min beyond the cessation of tDCS. The sham group had 30 s of current ramping up to 2 mA and then backing down to 0 mA simultaneous with the start of language therapy. These procedures have successfully blinded participants to the stimulation condition (Gandiga et al., 2006), as well as the speech‐language therapist. The protocol required 15 consecutive weekday sessions for each participant. Efforts were made to adhere to the schedule, though some participants had to leave a few days earlier because of other commitments (median number of sessions: sham=11, tDCS=13). In the language therapy, we combined the spell‐study‐spell procedure with an oral and written naming paradigm and developed individualized trained and untrained word sets (Ficek et al., 2018), where trained and untrained sets (10–30 words depending on individual severity) were matched in length and frequency. Each participant was shown a picture on a computer, asked to orally name it, and to write the name. If the participant could not name the picture (orally or in writing), they were asked to provide three characteristics of the item to evaluate and reinforce semantic knowledge. If they still could not describe the word orally, they were offered the correct word and asked to repeat for three times. Likewise, if the participant could not write the word, or wrote it incorrectly, the therapist would offer the correct spelling in a spell–study–spell procedure. That is, the therapist wrote the correct word, reviewed each letter's sound, and then asked the participant to copy the word three times. Letter accuracy was determined based on a scoring system (Goodman & Caramazza, 1985) that considered letter deletions, additions, substitutions, and movements. Rather than whole‐word accuracy, letter accuracy was considered as a more precise evaluation as it captures the effects of different types of errors. Each letter was evaluated with 1 point, 0.5 points for correct identification, and 0.5 points for correct position. Scores for trained and untrained words were transformed to percentage points for each participant.

**TABLE 1:** Patient demographics

	Combined (*n* = 47)	tDCS (*n* = 25)	Sham (*n* = 22)
Sex	22F, 25M	11F, 14M	11F, 11M
PPA variant	15L, 23N, 9S	9L, 12N, 4S	6L, 11N, 5S
Age	67.3 (6.8)	65.8 (8.1)	69.1 (5.0)
Year post onset	4.2 (2.8)	4.3 (3.2)	4.0 (2.3)
Language severity	1.7 (0.8)	1.7 (0.9)	1.8 (0.8)
Total severity	6.3 (4.5)	5.7 (3.9)	7.0 (5.2)

*Note*: For age, years post‐onset, severity, values shown are mean (SD). The *p*‐values are from the Welch two sample *t*‐tests for continuous outcomes and Fisher's exact test for categorical outcomes. Language severity is based on the language subset from the FTD‐CDR scale. Total severity refers to the sum of boxes, including language and behavior as added in Knopman et al. (2008).

A total of 50 right‐handed, native English‐speaking patients had a pre‐intervention scan (scan1) and 48 had a post‐intervention scan (scan2). One patient was deleted from the analysis because of missing values in the connectivity matrix. Among the remaining 47 post‐intervention scanned patients, 25 had transcranial direct‐current stimulation + language therapy and the remaining 22 patients had the sham treatment plus language therapy. Several baseline covariates were recorded including gender, disease onset (years), age at the start of therapy, and language severity. These patients were diagnosed with three variant types, including: logopenic, nonfluent, and semantic. Diagnoses were based on which function(s) were compromised. Patients with the *Logopenic* variant PPA (lvPPA) present with word‐finding difficulties and disproportionately impaired sentence repetition. Patients with *nonfluent* variant PPA (nfvPPA) present with difficulty producing grammatical sentences and/or exhibit motor speech impairment (apraxia of speech). Finally, patients with *semantic* variant PPA (svPPA) present with fluent speech, but impaired word comprehension. See Table 1 for a summary of demographic and clinical information on the participants.

### 2.2 Data preprocessing

MRI scans were obtained at the Kennedy Krieger Institute at Johns Hopkins University, using a 3 T Philips Achieva MRI scanner equipped with a 32‐channel head coil. Resting‐state fMRI (rsfMRI) data were acquired for ~9 min (210 time‐point acquisitions) post‐intervention. We used a 2D EPI sequence with SENSE partial‐parallel imaging acceleration to obtain an in‐plane resolution of 3.3 × 3.3 mm^2^ (64 × 64 voxels; TR/TE = 2500/30 ms; flip angle = 75°; SENSE acceleration factor = 2; SPIR for fat suppression, 3‐mm slice thickness). The data were co‐registered with structural scans into the same anatomical space. Structural scans, acquired axially with a scan time of 6 min (150 slices), used a T1‐weighted MPRAGE sequence with 3D inversion recovery, magnetization‐prepared rapid gradient, isotropic with a resolution of 1 × 1 × 1 mm^3^ (FOV = 224 × 224 mm^2^; TR/TE = 8.1/3.7 ms; flip angle = 8°; SENSE acceleration factor = 2).

Using MRICloud, a cloud‐platform for automated image parcellation approach (atlas‐based analysis), the MPRAGE scan was parcellated into 283 structures (Mori et al., 2016). In detail, each participant's high‐resolution MPRAGE was segmented by using a multi‐atlas fusion label algorithm (MALF) and large deformation diffeomorphic metric mapping, LDDMM (Ceritoglu et al., 2013; Miller et al., 2005; Tang et al., 2013). This highly accurate diffeomorphic algorithm, associated with multiple atlases, minimizes the mapping inaccuracies due to atrophy or local shape deformations. All analyses were performed in native space. To control for relative regional atrophy, volumes for each ROI were normalized by the total intracerebral volume (total brain tissue without myelencephalon and cerebrospinal fluid). The resting‐state fMRI was also processed in MRICloud and analyzed in a seed‐by‐seed manner. Image processing is described in Faria et al. (2012) including routines imported from the SPM connectivity toolbox for coregistration, motion, and slice timing correction, physiological nuisance correction using CompCor (Behzadi et al., 2007), and motion and intensity TR outlier rejection using ART (https://www.nitrc.org/projects/artifact_detect/). The MRICloud pipeline followed established steps for rsfMRI processing as follows. After exclusion of outlier TRs per the ART routine (parameters: 2 SDs for motion and 4 SDs for intensity, more severe than the default of 9), the movement matrix combined with the physiological nuisance matrix was used in the deconvolution regression for the remaining TRs. Outlier rejection and regression of motion parameters minimize potential motion effects. The parcels resulting from the high‐resolution T1 segmentation were brought to the resting state dynamics by co‐registration. Time‐courses of 78 cortical and deep gray matter ROIs were extracted and the correlations among them were calculated.

### 2.3 Density regression

We propose to quantify the effect of possibly non‐localized stimulation on functional connectivity through a density regression. Let $C_{i}\left(u,v\right)$ be a connectivity measure, such as the correlation of the BOLD time series, between foci u and v for u=1…p and v=u…p and then let $C_{i}$ be the collection of connectivity measurements, typically represented by a symmetric matrix, but in our case simply an ordered vector. We study the distributional summary of the collections of $C_{i}$ exactly as if they were drawn independently from a distribution. Let ${\hat{f}}_{i}$ be the estimate of the associated density $f_{i}$ of connections for subject i. Our proposal is to analyze $f_{i}$ with functional regression methods. A motivation for studying $f_{i}$ can be obtained by the weaker assumption of exchangeability of the labels. Such exchangeability translates in this context to the relevant information for predicting the outcome being in the proportion of stronger and weaker connections, regardless of where they occur.

**FIGURE 1:**
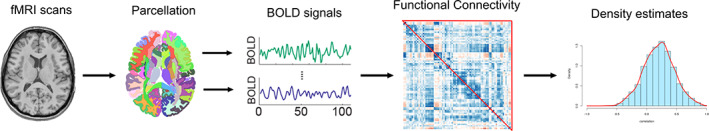
From MRI scan to connectivity density.

The process of proceeding from fMRI scans to the connectivity density is outlined in Figure 1. We estimated the connectivity matrix via temporal correlations of BOLD signals between ROIs after parcellation, which were then passed to a density estimation algorithm. Specifically, we used the vectorized elements in the upper triangular portion of the connectivity matrix to estimate the density using smoothing splines (Gu & Qiu, 1993). This performs maximum likelihood estimation on the spline coefficients for estimating the logarithm of the density function under a smoothness penalty. We chose this approach as it directly returns the splines, which are both mathematically and practically convenient, especially for performing a functional regression. In addition, it sets a boundary on the support for the estimated density, which is beneficial here, as correlation coefficients are bounded between −1 and 1. Kernel density estimators (Silverman, 1986) were also implemented as a comparison.

Our proposal is to use ${\hat{f}}_{i}$ to characterize $C_{i}$ and subsequently study the relationship between ${\hat{f}}_{i}$ and variables of interest. In the tDCS study, the variable of interest is treatment status. Since the $\left\{{\hat{f}}_{i}\right\}$ are (infinite dimensional) functional data, we employ functional data analysis tools (McLean et al., 2014; Ramsay, 2004; Ramsay & Silverman, 2007). Logically, one would model that treatment status predicts connectivity. However, treating complex data as covariates is typically more convenient than treating them as the outcomes. For example, the ability to incorporate other covariates is simply adding terms in a regression model. Unlike models for complex multivariate structured outcomes, an outcome reversed functional approach can be easily implemented with existing software tools available in any statistical package. As such, the method extends easily to longitudinal models, whereas longitudinal models for complex structured outcomes are not fully developed. Putting connectivity densities as covariates also makes the method directly extendable to predicting subject‐specific behavior scores. Therefore, we adopt the ideas in case–control inverse regression (Prentice & Pyke, 1979; Rothman et al., 2008), and predict whether a subject is in the treatment arm using the connectivity density and the baseline covariates as predictors. Let $A_{i}$ denote the treatment assignment with $A_{i}=1$ for tDCS and $A_{i}=0$ for sham, and $X_{i}\in {\mathbb{R}}^{q}$ denote the *q*‐dimensional covariate vector with the first element one for the intercept. The linear model considered is the following:

(1) $$\text{logit}\left\{P\left(A_{i}=1|X_{i},f_{i}\right)\right\}=X_{i}^{Τ}\beta +\int T\left({\hat{f}}_{i}\right)g,$$

where T is a given operator from ${ℒ}^{2}$ to ${ℒ}^{2}$ aiming to capture a specific characteristic of the density functions. T can also be used to control the impact of possible outliers of connectivity measures, such as using quantile‐based transformations. The function g is a coefficient function representing the effect of the tDCS used in this experiment, which can potentially change for different simulation settings. The parameter $\beta \in {\mathbb{R}}^{q}$ is the coefficient vector of the covariates, both to be estimated.

Various choices of T and the shape of g have different interpretations on the resulting model. For example, setting $T\left(f\right)=f$, the identity function, the linear predictor is $\int T\left(f_{i}\right)g=E\left[g\left(Z_{i}\right)\right]$, where $E\left[\cdot \right]$ is the expectation of a random variable and $Z_{i}$ is a random variable drawn from $f_{i}$. With a sufficiently flexible choice of g, Model (1) covers a broad range of possible model fits. However, many of them may not focus on non‐central components of the density, where effects would likely occur because of the stimulation procedure. For example, if g is a polynomial, the model considers the moments of the density (mean, variance, skewness, etc.) as predictors. However, it offers no benefit over the direct usage of the moment estimates of the connectivities. Thus, polynomial bases will not be discussed further, though they do demonstrate an interesting special case of the approach.

As for the choice of T, using $T\left(f\right)=\log\left(f\right)$ is similar to the use of the identity function. It loses the expected value interpretation, while instead, performs regression on the space of densities with Aitchison geometry (Egozcue et al., 2006). Thus, it may better detect the influence of the tail behavior on the outcome.

**FIGURE 2:**
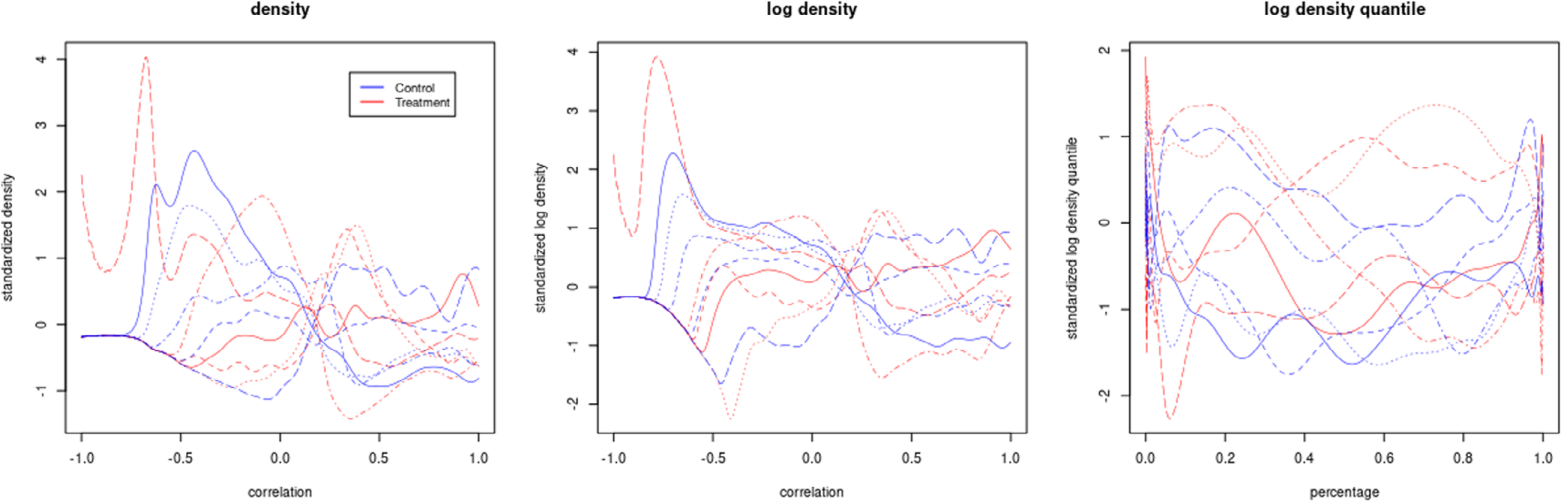
An illustration of connectivity densities, its log transformation and its log density quantiles. Plots shown for 10 random sampled subjects in our tDCS study and functions are standardized across all subjects to have similar y scales along *x*‐axis.

Another choice is the quantile mapping, $T_{q}\left(f\right)=F^{-1}$, where F is the cumulative distribution function associated with the density f. With a sufficient number of foci, this approach is approximately equivalent to using the empirical quantiles of the connectivity data as the regressors. Our proposed approach is quite similar to this. However, we further propose to weight the quantiles via density quantile. Specifically, we set $T_{\mathrm{ldq}}\left(f\right)=\log\circ f\circ F^{-1}=-\log\left[\left({\mathrm{dF}}^{-1}/\mathrm{dt}\right)\right]$ where ∘ is the function composition operator. The latter equality is easy to derive by taking derivatives via the chain rule to the identity function, $F\circ F^{-1}$. Note that the density quantile $f\circ F^{-1}$ can be regarded as a quantile synchronized version of the density function, and therefore is more sensitive to the changing tails. The logarithm transforms maps density quantile to a Hilbert space, which is practically useful for linear models. This idea has been explored before as a potentially preferable method for utilizing quantiles as regressors. Specifically, it is equivalent to the Hilbert space mapping, suggested by Petersen and Muüller (2016). Figure 2 shows original densities, log transformed densities and log density quantiles of 10 random sampled subjects in our tDCS study.

### 2.4 Reversing the predictor/response relationship

It is typical in regression models to consider the hypothetically functionally antecedent variable as a predictor, independent or exogenous variable, rather than an outcome, dependent or endogenous variable. A counterexample is in outcome‐dependent sampling, such as in retrospective studies. We utilize the same strategy of reversing the typical predictor/response relationship, as is more convenient for modeling with high dimensional and complex quantities (such as brain connectivity) as the predictor. In the tDCS study, we model treatment assignment as the outcome using a logit model with the connectivity density and other covariates as the independent variables. This avoids the need to construct probability distributions on the connectivity densities themselves.

To elaborate, using Bayes' rule and $P\left(A_{i}=1\right)=P\left(A_{i}=0\right)=0.5$ (due to the randomization), for any function g and transformation T, we have:

$$\text{Odds}\left(A_{i}=1|X_{i},\left\langle T\left(f_{i}\right),g\right\rangle \right)=\frac{P\left(\left\langle T\left(f_{i}\right),g\right\rangle |A_{i}=1,X_{i}\right)}{P\left(\left\langle T\left(f_{i}\right),g\right\rangle |A_{i}=0,X_{i}\right)},$$

where $\left\langle \cdot ,\cdot \right\rangle$ is any inner product of two functions. In our application, we consider logit models on $P\left(A_{i}=1|X_{i},T\left(f_{i}\right)\right)$, which depend on $f_{i}$ only though the form $\left\langle T\left(f_{i}\right),g\right\rangle$. Also, thanks to the randomized design, we can be aggressive in excluding potential confounders as covariates. This is especially helpful given the modest sample size. As the above relationship shows, our treatment assignment outcome model, $P\left(A_{i}|X_{i},T\left(f_{i}\right)\right)$, is consistent with any connectivity outcome model, $P\left(\left\langle T\left(f_{i}\right),g\right\rangle |A_{i},X_{i}\right)$, where the likelihood ratio comparing treated to controls is approximately log linear with our linear separable density model given in Equation (1).

### 2.5 Estimation of the coefficient function

To estimate the coefficient function, g in Model (1), we performed a functional principal components analysis (fPCA, see Reiss & Ogden, 2007, for a review). This reduces the dimension of the functional regressor using a set of data‐derived bases. In this approach, one calculates the PCA decomposition of the functions, $\left\{T\left({\hat{f}}_{i}\right)\right\}$, using the Karhunen/Loève transformation (Ghanem & Spanos, 2003), where the covariance function is smoothed (Di et al., 2009). We selected the leading principal components which explained over 99% of the variation as the basis functions. Notice that the version of fPCA utilized here does not honor possible density implied constraints of $T\left({\hat{f}}_{i}\right)$. Generalized cross‐validation (GCV) was used to choose the smoothing parameters (for detailed discussion, see section 4.5.4 of Wood, 2004). Confidence bands were derived using a Bayes approach (McLean et al., 2014; Nychka, 1988; Wahba, 1983).

### 2.6 Comparison

To illustrate the benefit of conducting a delocalized analysis, a simulation study based on the fMRI data collected in the tDCS study was conducted. We demonstrate an extreme example where non‐localized brain stimulation decreases statistical power, or even makes it impossible to identify ROI pairs with a significant effect when implementing a localization method. However, using connectivity densities retains the relevant information. Our goal in this simulation was to create a caricature of non‐localized effects, to demonstrate the statistical direction that the procedure highlights.

As a correlation coefficient, connectivity can be written as $\cos\left(\theta \right)$ where θ is the angle between two signals. In the simulation, consider a brain connectivity map with 20 regions, $R_{1}\ldots R_{20}$. For every map, let $\theta_{\mathrm{ij}}$ be the angle between signals in location i and j; we simplified the data generating distribution by assuming that the angles, $\theta_{\mathrm{ij}}$, are i.i.d. following a von‐Mises distribution, $M\left(\mu ,k\right)$, where the density is $f\left(\theta |\mu ,k\right)=e^{k\cos\left(x-\mu \right)}/2\pi I_{0}\left(k\right)$, with $I_{0}$ as the modified Bessel function of order 0. The parameters, μ, k were estimated from pre‐intervention patients by maximum likelihood. This was done to have a realistic null distribution on densities.

A non‐localized “stimulation” was simulated by perturbing region $R_{i}$ with equal probability across i. After stimulation, we simplified the effect via a degree rotation, ϕ, for the signal at $R_{i}$. Correspondingly, all $\theta_{\mathrm{ij}}$ change the same amount and the final post‐stimulation connectivity was a convex combination of this stimulated matrix and the pre‐stimulation matrix, where the weight was used to control the signal level and therefore controls the degree of difficulty in detecting the effect. Denote $C_{\mathrm{ij}}$ the pre‐stimulation correlation between region i and j, that $C_{\mathrm{ij}}=\cos\left(\theta_{\mathrm{ij}}\right)$. A stimulation on region $i_{0}$ yield a symmetric post‐stimulation connectivity $C_{\mathrm{ij}}^{\mathrm{sti}}$ as

$$C_{\mathrm{ij}}^{\mathrm{sti}}=\left(\begin{array}{cc} C_{\mathrm{ij}} & i\neq i_{0},j>i\\ {\mathrm{wC}}_{\mathrm{ij}}+\left(1-w\right)\cos\left(\theta_{\mathrm{ij}}+\phi \right) & i=i_{0},j>i \end{array}\right.$$

Notice that, although uniform stimulation on all regions of $R_{i}$ is unpractical in many situations, this simulation is a boundary case to understand the effect of lacking localization. Mover, it is still consistent with other kinds of non‐localized effects that are random mixture of localized effects. In Appendix A, we also describe and examine another intuitive simulation setting, and we observed similar results.

For every run of the simulation, we sampled 50 pre‐stimulation maps from the pre‐intervention scans and fit the parameters μ,k for each. We subsequently simulated 50 connectivity maps from samples of fitted von‐Mises distributions, and applied the stimulation above for a random half of these maps. We chose ϕ=π, and the weight w in the convex combination was chosen to be 75%. Other values, ranging from 90% to 50%, were also tried and similar patterns were observed. Weights under 50% made the signal detection too easy and methods are indistinguishable. Significance results for edgewise testing, principal component regression, and density regression were compared, with different density regression transformations for 1000 simulations. For completeness, we also considered instances with no stimulation effect and when the stimulation was localized at a specific region.

The edgewise regression approach considers the following model:

(2) $$\text{logit}\left\{P\left(A_{i}=1|X_{i},f_{i}\right)\right\}=X_{i}^{Τ}\beta +C_{i}\left(s,t\right)\alpha_{\mathrm{st}},$$

where s>t. The second approach was a regression model with dimension reduced predictors:

(3) $$\text{logit}\left\{P\left(A_{i}=1|X_{i},f_{i}\right)\right\}=X_{i}^{Τ}\beta +S_{i}\alpha ,$$

where, $S_{i}$ are the leading principal components of the vectorized connectivity matrix, $C_{i}$. We refer to this model as the PC model.

## 3 RESULTS

### 3.1 Simulation

**FIGURE 3:**
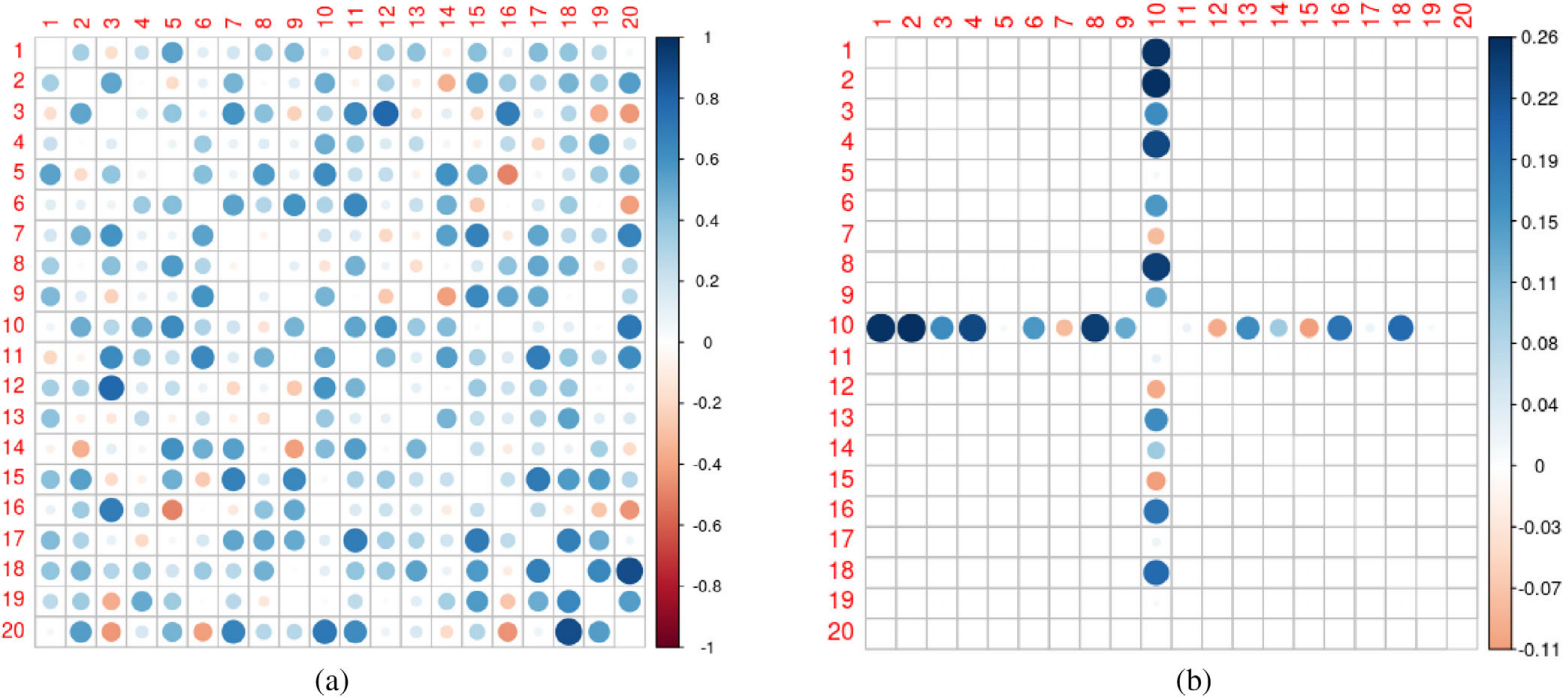
Part figure (a) shows the simulated pre‐stimulation connectivity matrix of a subject and part figure (b) is the simulated post‐pre difference in the connectivity matrix of the same subject.

**FIGURE 4:**
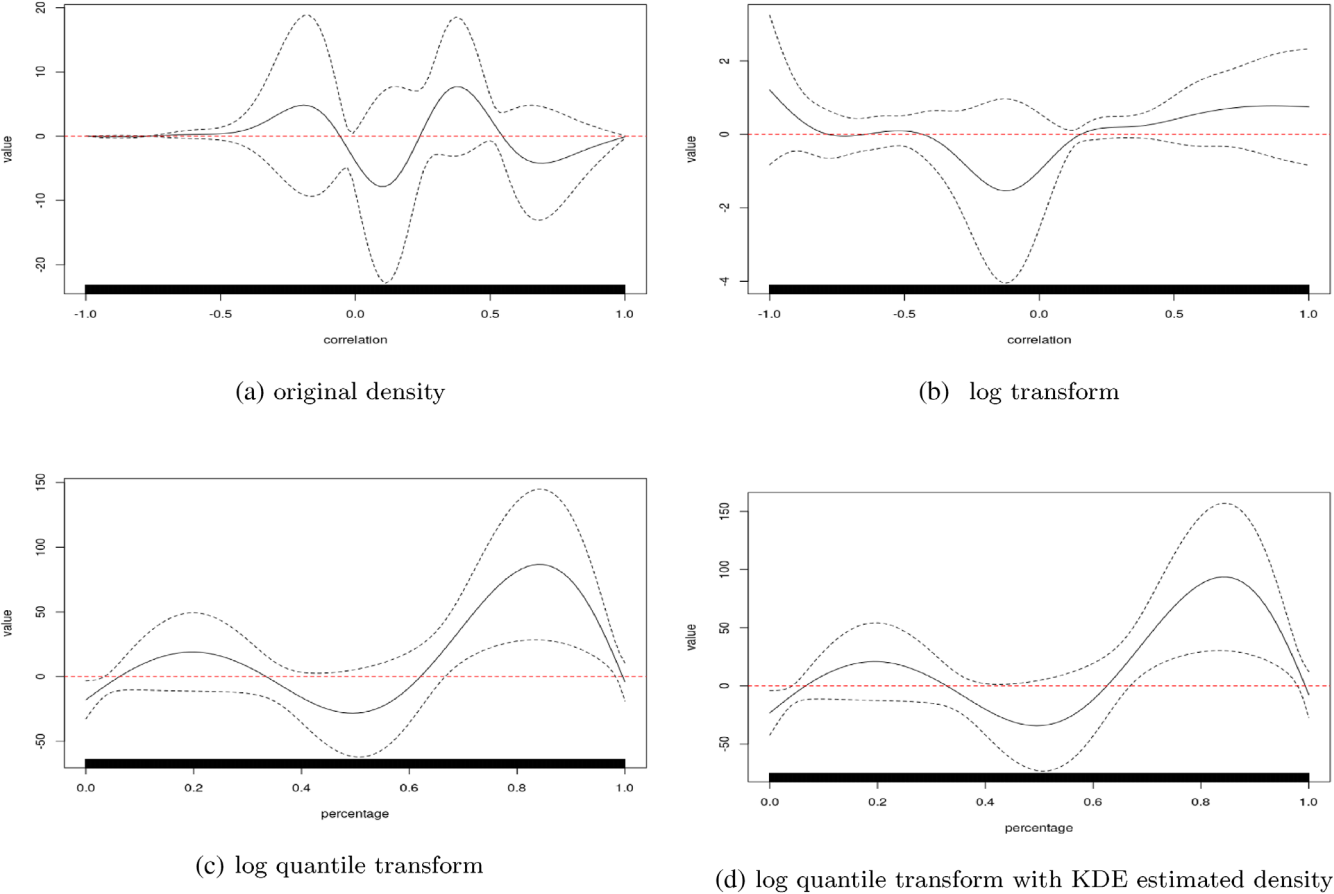
Model results on the tDCS experiment. The black solid line is the fitted coefficient function, g, with the black dashed line referencing the associated 95% confidence interval. Densities were estimated from smoothing splines implemented in the **fda** R‐package with 19 degrees of freedom for the spline basis. A kernel density estimator (KDE, d) is also computed and compared with smoothing spline (panel c) method. Contrasting c and d show that the density estimation technique did not impact results.

Figures 3a and 4a show example connectivity matrices and the difference after stimulation from an example simulation. The virtual stimulation was applied at region 10 in the right panel plot, while the left panel is the pre‐stimulation map. We report the rate of positive findings for all methods. Results are shown in Table A1. Localization methods, including the dimension reduction method, do not find any significant region pairs in the non‐localized simulations. In contrast, in this setting, the density method detected the stimulation impact on the connectivity densities. Among all the transformations, the log density‐quantile transformation was significantly better than others. We would like to reiterate that the simulation is contrived to highlight an extreme setting. Connectivity density methods will not necessarily increase the sensitivity of the analysis. If the true effect is localized, it cannot be better than well‐specified localized method.

### 3.2 Analysis of the tDCS data using localized methods

For the tDCS data, we tested the significance of the edgewise regression [Model (2)], a principal components regression [Model (3)] and a LASSO post‐inference model (Dezeure et al., 2015) using connectivity of all ROI pairs. No foci‐pair or principal components was identified as significant in either regression model, at Type I error rate levels of 0.05 or even 0.1. Of note, previous localization work on related data (Ficek et al., 2018), yielded significant findings. However, the total number of regions were restricted, thus dramatically reducing multiplicity concerns. In this analysis, 78 regions were used, resulting in a more stringent correction factor based on 78 choose 2, or 3003 comparisons. In addition, a more restrictive inclusion criteria in Ficek et al. (2018) led to a different study population.

### 3.3 Analysis of tDCS data using density regression

In this section, we present the analysis results of the tDCS study using the density regression Model (1) with different transformations (T). The fitted coefficient function, g, and its 95% confidence interval are presented in Figure 4. Functional linear regression was performed using the refund R‐package with default parameter of smoothed covariance fPCA, which chooses the number of components that explains over 99% of the data variation.

Regressing on the density after applying the log‐density quantile transform yielded the highest number of significant signals, which reaches its maximum around the 85th percentile. This potentially indicates that stimulation has a consistent tail effect, which is more likely to be aligned by quantile, rather than absolute value. Since the estimated coefficient function is significantly non‐zero only in the positive tail this suggests that the tDCS group had higher connection densities in the tail than the sham group. That is, connectivity among the most connected regions was higher in the tDCS group.

A likelihood ratio test was performed to compare logistic regression with only baseline variables and our model including both the baseline variables and the log density quantile term. The resulting *p*‐value was .0052, indicating a statistically significant gain of information from connectivity density at the 0.05 benchmark type I error rate. The conclusion remains true if one applies a Bonferroni *p*‐value correction. Specifically, three transformations were compared and therefore the corrected *p*‐value is .017. Notice that this is already a conservative value. The result agrees with a non‐parametric permutation test where we do the same regression but connectivity densities of subjects are randomly shuffled. Using AUC as test statistic, we observe that the AUC of log density quantile model is also significant larger than that of null distribution, which is the AUCs with shuffled connectivity densities. The *p*‐value is .015±.0009 estimated from 20,000 runs. A further reanalysis of subgroups shows that the effect is driven primarily by the *nonfluent* subtype which comprises 23 over total 47 subjects. There is not enough data to investigate the possibility of different effects of other subtypes, the least of which only has 9 subjects. We also performed a sensitivity analysis examining the impact of hyperparameters in the density estimation. We changed the smoothing parameter in spline smoothing and bandwidth in kernel density estimation method, both in the range of $\left[\theta_{0}/2,2\theta_{0}\right]$, where $\theta_{0}$ is the corresponding default value. For smoothing splines this value was selected by the approximated cross‐validation method suggested in Gu and Wang (2003) and for KDE this value is suggested by Silverman (1986). We observed that the log density quantile transformed model constantly gives significant information gain with *p*‐value <.05 in all settings, comparing with the demographic‐only baseline model. Therefore, the method is not sensitive to reasonable deviations in hyperparameter selection.

We also studied the effect of the estimated function on behavior change. We found that the variable $\int T\left({\hat{f}}_{i}\right)\hat{\beta }$ is significant (*p* < .05) for predicting the change of language ability, measured by untrained items, after transcranial direct‐current stimulation. Here $\hat{\beta }$ is the coefficient function estimated above for $T=T_{\mathrm{ldq}}$ and, recall, ${\hat{f}}_{i}$ are the connectivity densities for post‐intervention scans. The result shows a necessary condition for connectivity density mediating the effect of stimulation on language ability, which can motivate a future formal mediation analysis.

### 3.4 Induced connectivity

Consider the best model using the log density quantile transform, $T_{\mathrm{ldq}}$. We have

$$\text{logit}\left\{P\left(A_{i}=1|X_{i},f_{i}\right)\right\}=X_{i}^{Τ}\beta +\int_{0}^{1}\log\left[f_{i}\circ F_{i}^{-1}\left(q\right)\right]g\left(q\right)\mathrm{dq}.$$

Notice that for the connectivity matrix, $C_{i}$, we have $F_{i}\left\{C_{i}\right\}∼U\left(0,1\right)$, a uniform distribution on $\left[0,1\right]$ via the probability integral transform. Let $Q_{i}\left(s,t\right)=F_{i}\left\{C_{i}\left(s,t\right)\right\}$. Then, it follows that:

$$\int_{0}^{1}\log\left[f_{i}\left\{F_{i}^{-1}\left(q\right)\right\}\right]g\left(q\right)\mathrm{dq}=E\left[g\left(Q_{i}\right)\log f_{i}\left\{F_{i}^{-1}\left(Q_{i}\right)\right\}\right]$$

$$≍\frac{2}{N\left(N-1\right)}\sum_{t>s}g\left\{Q_{i}\left(s,t\right)\right\}\log f_{i}\left[F_{i}^{-1}\left\{Q_{i}\left(s,t\right)\right\}\right].$$

**FIGURE 5:**
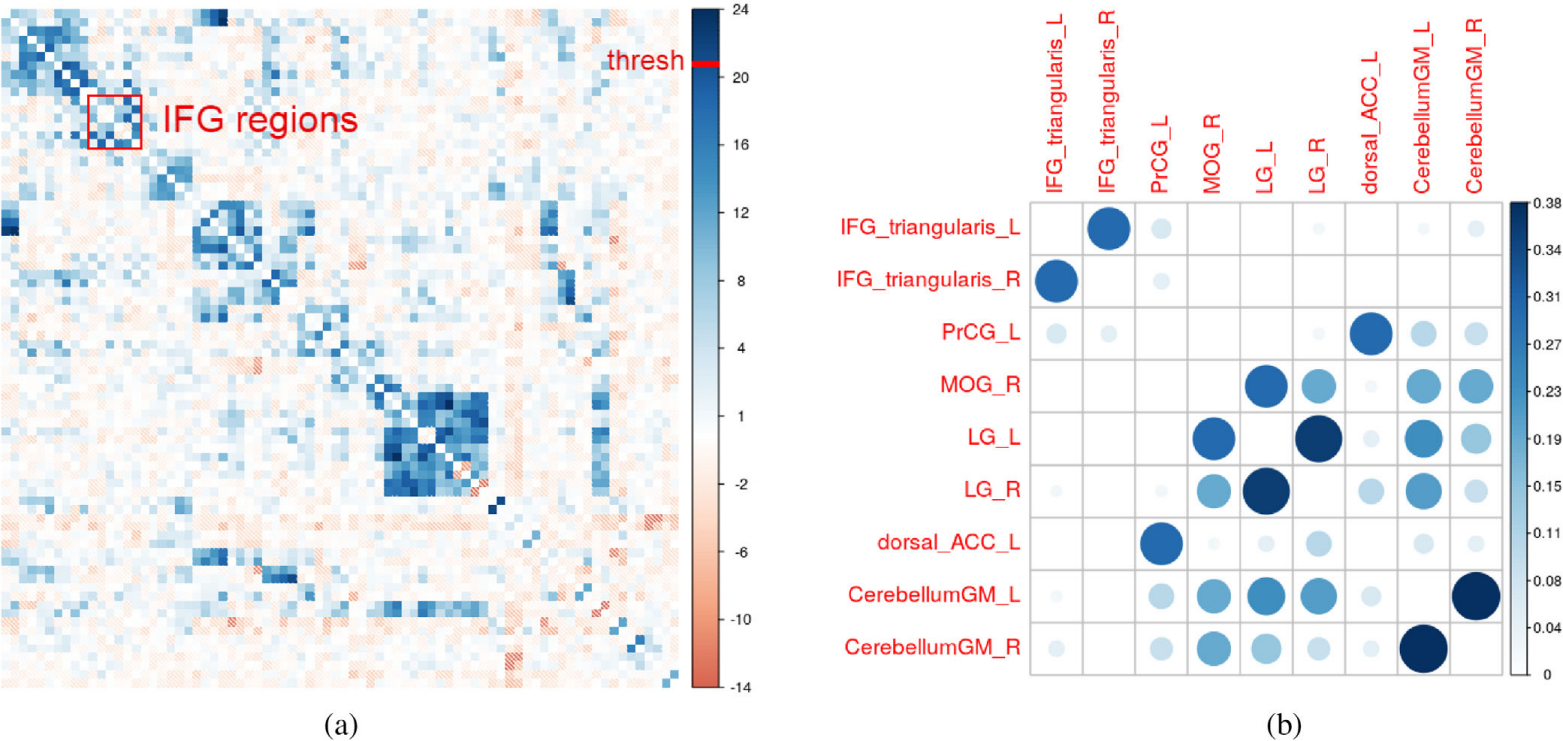
Part figure (a) shows the induced connectivity described in Section 3.4. IFG regions (the tDCS target) are noted in the red box. Part figure (b) shows some region pairs with the most consistent contribution, measured by the frequency of having top 5% absolute effect size across all patients.

Therefore, for this subject, one can assign $g\left\{Q_{i}\left(s,t\right)\right\}\log f_{i}\left[F_{i}^{-1}\left\{Q_{i}\left(s,t\right)\right\}\right]$ as the effect size for region pair $\left(s,t\right)$. Averaging this effect across all patients yields an important metric for every region pair in the model. We call this stimulation‐induced connectivity, since it describes how influential the correlation of each region pair is in predicting stimulation status. The induced connectivity matrix is shown in Figure 5, together with a summary of effect agreement across subjects, where for each patient, region pairs are selected with top 5% absolute effect size and the frequency of each region pair being selected is calculated.

This technique, of course, returns to a discussion of localized effects. However, by investigating this measure one can ascertain the degree of localization consistency across subjects—an impossibility with pure localization analysis.

## 4 DISCUSSION

In this manuscript, a new framework for analyzing functional connectivity was explored. Functional data analysis of log quantile connectivity densities investigates possible nonlocalized effects associated with subject‐level variables. It is clear that our method can be directly applied to other kinds of numerical measurements. For example, partial correlations or entropy‐based measures. However, it continues to be only useful suitable if connection exchangeability represents a useful model. A sizable by‐product of this style of analysis is the drastic reduction of multiplicity considerations. This is of great importance in connectivity analysis, where the number of comparisons grows at a rate of the square of the number of foci being considered. In the data application, we find associations between stimulation and connectivity density. In contrast, edgewise methods fail to find any results, because of multiplicity issues. This is partially due to a wide search of all possible region pairs from the parcellation. Of course, one could also reduce multiplicity concerns by restricting attention to regions associated with a priori hypotheses of interest, as was done in Ficek et al. (2018). In contrast, investigating connection densities is an omnibus approach that benefits from a reduction in the number of tests over exploratory edge‐wise approaches, a robustness to non‐localized effects and a robustness to the inclusion of unnecessary foci. These benefits come at the expense of the loss of power and interpretability over analyses considering only a small set of tightly specified edge‐wise hypotheses. Our method can also be extended to seed‐based connectivity and voxel‐by‐voxel connectivity without any modification. However, the assumption of complete node invariance discards a potential sizable amount of relevant localization information. Therefore, we believe that the method would be primarily useful as an easy and simple early‐stage omnibus test, or after light localization efforts, such as considering connectivity densities between voxels within sets of ROI. To further emphasize the ease and simplicity of the method, we stress that density regression can be coded from scratch in only a few lines of code in any modern scripting environment with PCA and GLM functions.

Density regression, as a prediction model, can be view as a generalization of connectome‐based predictive modeling (Shen et al., 2017). Connectome‐based predictive modeling (CPM) uses individual connectivity matrices to predict behavioral measures. The method first selects location‐pairs that are most significantly correlated with the outcome, then summarizes the matrix by adding up connectivity measures in selected pairs, and this sum is used as a predictor in a regression model. In CPM, there is no localized effect and CPM can be viewed as a regression on connectivity density using only a constant basis. Here we generalize it by utilizing more distributional information.

An interesting direction to pursue with connectivity density methods is to consider potential robustness to spatial registration (Oliveira & Tavares, 2014). The connectivity density can relatively easily be shown to be invariant to relabeling and affine transformations (see Theorem 1 in the Appendix A). In contrast, localization methods heavily rely on both accurate registration and biological functional localization across subjects. Therefore, it is interesting to posit that density regression could be used after only mild affine registration efforts prior to the more time‐consuming non‐linear registration.

However, to reiterate, ignoring potentially useful localization information can reduce power and sensitivity. Surely, the optimal strategy removes subject‐specific artifacts and reduces the search space with—correct—strong a priori hypotheses and then tests only those edges. However, in the absence of this ideal case, one is often confronted with a massive unstructured search problem with localization analyses. In contrast, density regression is more akin to an omnibus *F*‐test, looking over a large range of edges, dramatically mitigating multiple comparison issues in the favor of testing one overview hypothesis, rather than a large collection of highly specific ones. Therefore, we suggest the method as an early‐stage tool in a neuroimaging data analyst's toolbox.

We used functional data analysis to relate connection densities to outcomes. Functional data analysis tools (Ramsay & Silverman, 2007) have grown to be quite flexible. Thus, density regression approaches can be relatively easily generalized to handle different settings, such as any typical statistical outcome model and longitudinal data. Also, density estimates may naturally make adjustments for missing data, in the form of missing foci, since the density can remain the same in some contexts. This has potential broad implications for the study of stroke and other diseases with abnormal brain pathology. Localization methods are not available if the ROI is damaged or missing. In contrast, density‐based methods are easy to apply. In addition, we used PCA on the log quantile densities as the basis for functional regression. The result is that the method can be applied using standard software without modification. Other bases and penalization strategies may improve the approach. In fact, the utility and application of functional regression in neuroimaging has been greatly improved via recent research efforts (e.g., see Goldsmith, Crainiceanu, et al., 2011, Goldsmith, Wand, & Crainiceanu, 2011, Goldsmith et al., 2012; Reiss et al., 2017).

Utilizing functional regression also has the benefit of producing more interpretable models as compared with machine learning approaches. However, this is achieved at a likely cost of prediction performance. It is possible that ML approaches could navigate the trade‐offs between localization and exchangeability non‐parametrically and possibly achieve better prediction performance. Thus, we view density regression as a parsimonious modeling choice rather than a method to optimize prediction performance.

Statistically, we assumed independence between subjects and relied on the randomization to invert the predictor/response relationship using logit models. This borrows techniques from case‐referent sampling from epidemiology dating back to the seminal work of Cornfield (see Breslow, 1996; Greenhouse, 1982, for overviews). A benefit of doing this is that it is generally easier to have the more complex variable as the predictor rather than a response. To elaborate, to have a density as an outcome, predictions from the model must be functions that are both positive and integrate to one. Most existing functional approaches, especially point‐wise ones, would satisfy neither criteria nor modeling distributional outcomes is an active area of statistical research. The probability space containing the outcome is necessarily a probability distribution on distributions, such as a Dirichlet process. While this is not a problem per se, it makes inference more technically challenging. In contrast, by conditioning on the density, as we have done, its distribution does not need to be modeled and the fitting and inference require little more than well‐known generalized linear model techniques. In Appendix 3 we further the discussion in comparison with function on scalar regression. It is seen that, with almost no effort, one obtains the use of easier models (GLMs) and appropriate inferences by reversing the relationship and the resulting estimates are similar to those of function on scalar regression. However, because the constraints are not accounted for in the function on scalar model, inferences remain in question.

Nonetheless, we reiterate that the use of connectivity density as a regressor remains useful, even if one prefers not to flip the predictor/response relationship. For example, in our tDCS example, connecting the connectivity density to behavioral outcomes would be relevant, where the natural predictor would be functional connectivity.

Independence between subjects was used for inference. We also used density estimates for connection densities, techniques that implicitly require sampling assumptions for theoretical convergence. However, we contend that connectivity densities are intrinsically of interest, and therefore no appeals to super‐population inference and sampling assumptions are needed for estimation. This is analogous to spatial group ICA, where productive estimates are obtained via independence assumptions on voxels over space, without a true sampling or super‐population model for inference (Calhoun et al., 2001). An interesting future direction of research would investigate dependencies between foci correlations.

Our recommended approach uses log quantile densities as the functional predictor, rather than the density, distribution function, or quantile function directly (Petersen & Muüller, 2016). This approach has convenient theoretical properties, but also the practical benefit of focusing attention on tail behavior, where effects are most likely to be seen. Utilizing the quantile density also creates robustness to irrelevant foci pairs being included in the analysis.

Our simulations and data results focus on settings that highlight the benefits of an omnibus density regression approach. In the simulations, we investigated a non‐localized caricature of typical effects. Similarly, in our data analysis, we performed no filtering of regions prior to analysis (thus magnifying multiple comparison concerns). It was shown in the simulation, that functional density regression approaches can find real non‐localized effects, whereas, as expected, edgewise methods do not find any. It should be emphasized that the performance of the density regression approach is invariant to the distribution of effects across subjects, whereas edgewise approaches become viable as the degree of localization increases.

In addition, the flexibility of the approach finds effects in the real data, even though there are a great deal of irrelevant connections (i.e., unnecessarily included region pairs) being studied. Edgewise and other regression approaches are highly sensitive to unnecessary null connections being included in the analysis. A benefit of the data being considered is the likely existence of an effect related to the stimulation. However, we emphasize that a single omnibus approach does not represent a full analysis of the data. We recommend this approach as a global analysis to be performed prior to edgewise or other localization methods. This mirrors the classic ANOVA (analysis of variance) approach of performing an overall F test before investigating pairs of explanatory factor levels. It would be most useful in exploratory model building where foci selection is not restrictive. In cases of tightly coupled statistical hypotheses involving relatively few regions or foci, density regression would not be needed or particularly helpful.

This methodology raises many avenues for future research. For example, one the idea of non‐localized effects in dynamic connectivity (Hutchison et al., 2013) via stochastic processes of connectivity densities (by time). In addition, there are multiple alternatives for densities estimated from correlation of each region pair for contralateral regions. Here, it should be acknowledged that there is strong homotopic correlations from symmetric regions. One should then deal with multivariate densities estimated from pairs of correlations. This same logic could be applied to geographically close regions and for instances with longitudinal scans. The connectivity density of spectral information (de Haan et al., 2012), like leading principal component scores, should also be studied to potentially extract relevant brain graph properties.

Finally, there is the role that connectivity‐density methods could play in fMRI analysis of subjects with missing brain tissue, such as studies of stroke or surgical interventions. Connectivity density methods may be resilient to the missing data impact of differential brain structure in a way that localization methods are not. In fact, it is interesting to conjecture what localization methods even mean in these settings where a subset of subjects are missing areas of localization. In contrast, density methods may provide a more robust and well‐defined methodology. It is worthy of note that components of graph methodology (Bullmore & Sporns, 2009; Sporns, 2010) often considers summary metrics that do not require or assume localization. Density regression can be considered a subset of weighted graph metric analysis.

## CONFLICT OF INTEREST

The authors declare that there is no conflict of interest.

## Figures and Tables

**FIGURE A1:**
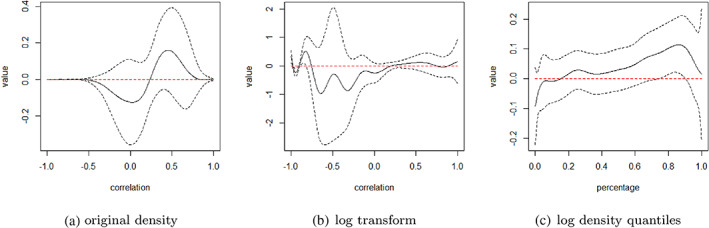
Estimated difference function of the transformed neural densities between treatment and control groups, holding all other variables the same. Similar patterns could be found compared with Figure 4a–d but their confidence bands are biased because no constraints on the outcome function are satisfied.

**FIGURE A2:**
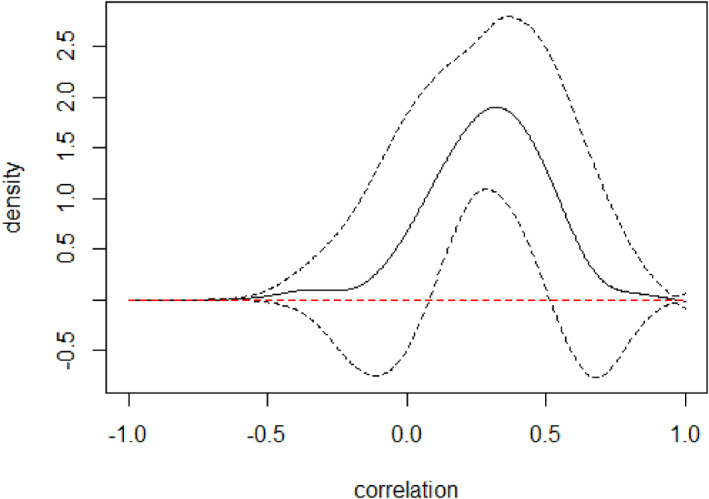
A sample outcome function from the fitted model. It breaks the positive constraints on both tails and its integral is 0.99<1. Also the confidence band from the model does not make sense because all densities are non‐negative.

## Data Availability

Data sharing agreement: We will make the data and associated documentation available to ;users only under a data‐sharing agreement that provides for: (1) a commitment to using the ;data only for research purposes and not to identify any individual participant; (2) a commitment ;to securing the data using appropriate computer technology; (3) proper acknowledgment of data ;source; and (4) a commitment to destroying or returning the data after analyses are completed. Data requests should be sent to tsapkini@jhmi.edu.

## APPENDIX A

### A.1 Invariance properties

Here we discuss some invariance properties of the connectivity density. Consider C a connectivity measure where $C\left(x,y\right)$ is measuring the connectivity between location x∈D and y∈D. The connectivity density can be defined as the density of random variables $C\left(U,V\right)$, where $\left(U,V\right)$ follows a sampling distribution on D×D. Denote $f_{\text{sample}}$ as the density of that sampling distribution. It is easy to see that the connectivity density, f, defined in Section 2.3 also follows such a definition while using the uniform distribution as $f_{\text{sample}}$. We prove that f is invariant to re‐labeling in discrete cases (e.g., connectivity between ROIs) and to affine transformation in continuous cases (e.g., interpolation of connectivity between voxels). Denote $\mathrm{supp}\left(C\right)$ be the support of connectivity measure C. After any invertible transformation, ℱ, the connectivity measure $C_{ℱ}$ will be naturally defined as $C_{ℱ}\left(x,y\right)=C\left({ℱ}^{-1}\left(x\right),{ℱ}^{-1}\left(y\right)\right)$. Then we have the following Theorem 1. Theorem 1
*Let* $\left(U^{C},V^{C}\right)$ *follow the uniform distribution on* $\mathrm{supp}\left(C\right)$ *. Then, the density of* $C\left(U^{C},V^{C}\right)$ *has the same distribution with* $C_{ℱ}\left(U^{C_{ℱ}},V^{C_{ℱ}}\right)$ *, where* ℱ *is any permutation map if* $\mathrm{supp}\left(C\right)$ *is a finite discrete set and* ℱ *is any affine transformation if* $\mathrm{supp}\left(C\right)$ *is a closure of some open set in* ${ℛ}^{3}$ *. Therefore, connectivity densities are invariant to these transformations*.

*Proof*. By change of variable calculus of random variables, we know that under a sampling distribution $f_{\text{sample}}$, $C\left(U,V\right)$ has the sample distribution with $C_{ℱ}\left(U^{\prime },V^{\prime }\right)$ if $\left(U^{\prime },V^{\prime }\right)∼f_{\text{sample}}^{\prime }$ where $f_{\text{sample}}^{\prime }\left(x,y\right)={\left|ℱ\right|}^{2}f_{\text{sample}}\left({ℱ}^{-1}\left(x\right),{ℱ}^{-1}\left(y\right)\right)$.

In our uniform cases, the Jacobian ∣ℱ∣ and sampling distribution $f_{U^{C},V^{C}},f_{U^{C_{ℱ}},V^{C_{ℱ}}}$ will always be a constant. Therefore, the condition above always holds and $C\left(U^{C},V^{C}\right)$ must follow a sample distribution with $C_{ℱ}\left(U^{C_{ℱ}},V^{C_{ℱ}}\right)$.

Since the uniform distribution is the only distribution invariant to all affine transformations / permutations, we know that the connectivity density defined in Section 2.3 is also the only possible distributional summary that has such an invariance property for arbitrary connectivity measures.

### A.2 Additional simulations

Here we describe another intuitive simulation setting and show that a similar pattern is observed. Specifically, it shows that our methods can detect non‐localized effects, while edgewise method or dimension reduction methods, like PCA, cannot, although the best transformation of densities might change for different signal distributions.

Again we consider connectivity matrix of 20 regions $R_{1},\cdots ,R_{20}$. A no‐stimulation connectivity matrix, C, is sampled uniformly from 50 pre‐intervention scans in our data and its 20 rows and columns are also uniformly sampled from an original 78 × 78 connectivity matrix. Now consider a localized stimulation as additive Gaussian signals to the Fisher‐*z* transformed correlation for specific region pairs. It then gives post‐stimulation connectivity matrix $C^{\mathrm{sti}}$ differs with C only on

(A1)
$$\tanh^{-1}C_{i_{k}j_{k}}^{\mathrm{sti}}=\tanh^{-1}C_{i_{k}j_{k}}+\varepsilon_{k}$$

for $\left\{\left(i_{k},j_{k}\right)|k=1,2\cdots K\right\}$ some specific region pairs and $\varepsilon_{i}$ i.n.d. follows $N\left(\mu_{k},\sigma_{k}^{2}\right)$. Notice that this formulation corresponds with the underlying effect pattern in some common edgewise analysis of change in connectivity, for example Ficek et al. (2018).

In the simulation for localized analysis, the locations $\left(i_{k},j_{k}\right)$ are uniformly randomly selected from all 190 region pairs and then fixed for all samples. Naturally a stimulation with non‐localized effect would also follow Equation (A1). But every time it is performed, $\left\{\left(i_{k},j_{k}\right)\right\}$ becomes another independent sample from the 190 regions. In the experiment, we choose $K=10,\mu_{k}=0.5,\sigma_{k}=0.5$ for all k. We also observed similar patterns for a variety of parameters settings. We ran the experiment for 10,000 independent simulations. For every run we sampled 100 no‐stimulation connectivity maps with another 100 each for localized stimulation, non‐localized stimulation and no stimulation. We studied how different methods work in these situations as described in Section 2.6. The results for the simulation can be found in Table A1. We observe a similar pattern as Table A2 that connectivity density based methods can detect non‐localized effect while edgewise analysis and principal component analysis cannot. It also shows that the optimal transformation might be different for different patterns of the effect, as the log transformation is the best in this situation while the log‐density‐quantile transformation is the best in Table A2.

**TABLE A1:** The table shows the ratio of significant positive findings over 10,000 runs

	Bonferroni	FDR	PC	$T_{0}$	$T_{l}$	$T_{\mathrm{ldq}}$
Non‐localized	0.060	0.065	0.089	0.618	1	0.870
Localized	0.938	0.953	0.994	0.620	1	0.862
No‐stimulation	0.048	0.051	0.082	0.060	0.056	0.053

*Note*: $T_{0}$, $T_{l}$, and $T_{\mathrm{ldq}}$ are density regressions with the identity, logarithm, and log density‐quantile transformations described in Section 2.3. Bonferroni, FDR (Benjamini & Hochberg, 1995) refer to edgewise regression with those associated multiplicity correction procedures. PC refers to principal component regression with the top 20 components.

**TABLE A2:** Significant positive findings over 1000 runs. T0,Tl,Tldq are density regressions with the identity, logarithm, and log density‐quantile transformation described in Section 2.3. Bonferroni, FDR (Benjamini & Hochberg, 1995) refer to edgewise regression with different multiplicity correction procedures. PC refers to the principal component regression with the top 10 components, the number chosen by minimizing the sum of type I error (significance ratio in non‐localized situation) and type II error (none significance ratio in localized situation).

	Bonferroni	FDR	PC	$T_{0}$	$T_{l}$	$T_{\mathrm{ldq}}$
Non‐localized	0.073	0.078	0.118	0.638	0.117	0.717
Localized	0.638	0.669	0.754	0.629	0.112	0.714
No‐stimulation	0.061	0.065	0.113	0.075	0.058	0.059

### A.3 Connectivity density as outcome

In this section we detail why we reversing the predictor/response relationship is a compelling idea and thus compare the results with a typical function‐on‐scalar regression with connectivity densities as outcomes.

Excepting the convenience, as discussed in Section 4, the main reason for reversing the predictor/response is that typical function‐on‐scalar regression methods can not satisfy the integral constraints on the outcome, which are densities or isomorphic transformation of densities. Therefore, the specified distribution is not correct, creating concern regarding inferences.

Consider the following typical linear functional model with outcome function y and features x.

(A2)
$$y\left(t\right)=f_{0}\left(t\right)+x\cdot f\left(t\right)+\varepsilon \left(t\right)$$

where y is a density functions, log density function, or the log‐density‐quantile transformations. Recall, density functions must be both positive and integrate to 1. Log densities require integral of their exponential to be 1 and Log density quantiles require their corresponding quantiles be supported within [−1, 1], because they are quantiles of correlations. It is easy to see that within the linear functional framework 5, all these constraints cannot be translated into individual constraints on estimation of $f_{0},f$. Therefore, the model is specifying an easily demonstrably false distribution, resulting in possibly incorrect inferences even if estimation remains viable. Other methods exist to correct this problem, for example Szabó et al. (2016); Chen et al. (2021), but this is an active area of research and is thus challenging to implement for most practitioners.

In Figure A1, we show the estimation results of model 5 on our data as a reference. These are the slope functions of the treatment assignment variable, the estimated differences before and after tDCS stimulation. We used the regression methods described in Reiss et al. (2010) to solve the problem 5 and the penalty parameters selected by generalized cross validation. There is, as expected a high degree of similarity between the corresponding curves and those in Figure 4a–d. But, as we explained above, the distributional assumptions are questionable in this context and the confidence bands remain in question, and therefore we do not report such results in the main article. We also note the distinction in convenience, whereby we obtain similar estimates using only a GLM, perhaps the most standard statistical model.

Figure A2 shows one sample outcome function from the fitted model. We have checked that it breaks the positive constraints on both tails and its integral is 0.99<1. Also it is clear that the confidence band from the model does not make sense because all densities should be non‐negative. Therefore the inference results from the model 5 are wrong.
